# Recognized and Potentially New Biomarkers—Their Role in Diagnosis and Prognosis of Cardiovascular Disease

**DOI:** 10.3390/medicina57070701

**Published:** 2021-07-08

**Authors:** Weronika Bargieł, Katarzyna Cierpiszewska, Klara Maruszczak, Anna Pakuła, Dominika Szwankowska, Aleksandra Wrzesińska, Łukasz Gutowski, Dorota Formanowicz

**Affiliations:** 1 Faculty of Medicine, Poznan University of Medical Sciences, 60-812 Poznan, Poland; weronika.bargiel98@gmail.com (W.B.); k.cierpiszewska@onet.pl (K.C.); kara.metr@gmail.com (K.M.); ania.pakula98@gmail.com (A.P.); dominika.szwankowska@interia.pl (D.S.); ola.wrzesinska@op.pl (A.W.); 2 Department of Medical Chemistry and Laboratory Medicine, Poznan University of Medical Sciences, Rokietnicka 8, 60-806 Poznan, Poland; lgutowski@ump.edu.pl

**Keywords:** atherosclerosis, inflammation, oxidative stress, biomarkers, cardiovascular disease, hsCRP, Lp-PAL2, IL-6, miRNA, osteocalcin, angiogenin

## Abstract

Atherosclerosis and its consequences are the leading cause of mortality in the world. For this reason, we have reviewed atherosclerosis biomarkers and selected the most promising ones for review. We focused mainly on biomarkers related to inflammation and oxidative stress, such as the highly sensitive C-reactive protein (hs-CRP), interleukin 6 (IL-6), and lipoprotein-associated phospholipase A2 (Lp-PLA2). The microRNA (miRNA) and the usefulness of the bone mineralization, glucose, and lipid metabolism marker osteocalcin (OC) were also reviewed. The last biomarker we considered was angiogenin (ANG). Our review shows that due to the multifactorial nature of atherosclerosis, no single marker is known so far, the determination of which would unambiguously assess the severity of atherosclerosis and help without any doubt in the prognosis of cardiovascular risk.

## 1. Introduction

It is well-known that cardiovascular diseases (CVD) are the leading cause of death globally since the beginning of the 20th century. According to WHO data in 2017 only, coronary artery disease (CAD) was a cause of almost one in three (17.79 out of 56 million) deaths worldwide.

Although atherosclerosis is the main cause of CVD, the mechanisms behind atherosclerotic plaque formation are still not fully understood. We are now aware that this is a consequence of complex, often interdependent processes, among which inflammation, oxidative stress, and the body’s immune response play a key role [1]. Atherosclerotic plaque formation occurs in four main successive stages: (1) damage to endothelial cells with their dysfunction; (2) lipoprotein deposition and oxidation; (3) inflammatory process; (4) formation of a fibrous cap. It should be remembered that there are individual differences in the dynamics of plaque development, see [2,3], which are at least partly due to aging and comorbidities [4]. As a result of the complexity of this phenomenon, there is widespread agreement among researchers that determining the parameters of lipid metabolism is not enough to predict what is happening in the arterial subendothelial layer. Sometimes, we can even draw the wrong conclusions if we rely on them. For example, consider the JUPITER study, which revealed that men and women with low low-density lipoprotein cholesterol (LDL-C) but increased markers of local low-grade inflammation (high sensitive C-reactive protein (hs-CRP)) showed a significant cardiovascular risk. Participants who achieved hs-CRP less than 1 mg/L had a 79% reduction in vascular events and achieved hsCRP concentrations that were predictive of event rates irrespective of the lipid endpoint [5].

Keeping in mind that the development of atherosclerotic plaque formation stages is at least partly reversible, we reviewed some of the atherosclerosis biomarkers to discuss their ability to predict the development of CAD, giving patients and their doctors time to react.

The main limitation in identifying a universal biomarker of the inflammatory response, a key player process in atherosclerosis, is the variability of the metabolic stress response among patients and the multifaceted nature of the complex disorder itself.

Therefore, we recommend using more than one biomarker because there is still no one that fits all the patients and gives reliable results in every case. Our study reviewed biomarkers from different related processes to emphasize the complexity of forming atherosclerotic plaque and show future work directions to translate their role into clinical use. We included well-recognized biomarkers: (1) CRP, one of the acute-phase proteins produced mainly by the liver in response to low-grade inflammation underlying atherosclerosis; (2) interleukin 6 (IL-6); a pro-inflammatory cytokine secreted, among others, by tunica medias’ smooth muscle cells, whose leading role consists in activating the inflammatory and autoimmune processes; (3) Lp-PLA2, an enzyme associated with both traditional (cholesterol-linked) and novel (inflammatory) pathways of atherosclerosis, which is synthesized by inflammatory cells and bonded mainly to LDL-C, with a small fraction linked to high-density lipoprotein cholesterol (HDL-C). We have also included potentially new biomarkers, such as (4) miRNA regulating gene expression by silencing complementary to itself mRNA pieces, with overexpression related to the development of vascular changes and CVD; (5) osteocalcin (OC), which is a multifunctional hormone produced by osteoblasts whose action regulates mineralization, glucose, and lipid metabolism with a role in the process of vascular calcification and atherosclerosis; and (6) angiogenin, which is a protein involved in forming new blood vessels that interact with endothelium and smooth muscles, which possibly plays a role in destabilizing coronary plaque.

There is still debate about the need to include markers of atherosclerosis and inflammation in traditional cardiovascular risk assessment. The pathogenesis of atherosclerosis is a complex process involving so many molecules and pathways that it is impossible to present them all. In addition, not every particle meets the criteria of being a potential biomarker. A biomarker is a term that describes different types of objective indicators of health or disease. As technology advances, these indicators are becoming more and more precise. On the other hand, the detection of new biomarkers turns out to be a time-consuming and costly operation due to the complexity of the structure of potential markers, most often proteins, and difficulties in the reproducibility of the methods used for their determination.

In the article, we have selected those markers that cover the most critical stages of plaque development and appear to be essential for assessing the clinical consequences of plaque instability.

In this review, we systematically searched for relevant articles based on both our research experience and the following criteria: peer-reviewed articles published in English, terms (atherosclerosis) AND (cardiovascular) AND (biomarker) using PubMed Central, MEDLINE, and Science Direct. The list of all studies included were manually searched to identify literature that might deserve inclusion.

Six biomarkers were selected in this way, including three known and three new ones. Then, in order to identify important studies on these biomarkers, we performed a search based on these mentioned criteria, using the name of one of the six selected biomarkers instead of the term (biomarker). In addition, when selecting the appropriate publications, we relied on our knowledge of the issues raised; therefore, to the best of our knowledge, no known key aspect of the selected biomarkers was omitted by us.

## 2. Biomarkers

### 2.1. Recognized Biomarkers

#### 2.1.1. High Sensitivity C-Reactive Protein

C-reactive protein (CRP) is one of the essential positive acute-phase proteins usually present at negligible levels in the plasma. Its concentration increases after 24–48 h after acute inflammatory trauma up to 1000-fold at sites of infection or inflammation, reaching a maximum of 24–48 h. CRP is synthesized predominantly as a native pentameric CRP (pCRP) in the liver [6]. This isoform can irreversibly dissociate at sites of inflammation into five identical non-covalently linked subunits—monomeric CRP isoforms (mCRP). This dissociation is promoted by the pCRP binding to phosphocholine residues of lysophosphatidylcholines on the cell surface exposed by phospholipase A2 (PLA2), which is a biomarker of vascular inflammation [7]. IL-6, promoting CRP de novo synthesis, appears to be CRP primary regulator. In addition, IL-6 signaling can be enhanced by interleukin-1β (IL-1β) and, to a lesser extent, by the tumor necrosis factor-alpha (TNF-α) [6,7,8,9].

A growing body of research indicates that pCRP has both pro-inflammatory and anti-inflammatory effects in a context-dependent manner. In contrast, mCRP has a strong pro-inflammatory effect on endothelial cells and their progenitor cells, leukocytes and platelets. The latter CRP may even exacerbate the inflammatory response. The existence of two protein conformations may explain conflicting data on CRP properties; see for a review [8].

Until recently, the only known physiological function of CRP was to label cells to initiate their phagocytosis via activation of complement and elimination. However, it is known that vital cells with reduced energy supply are also marked, which is helpful for a classic wound. However, it turns out that such an action has the opposite effect on internal injuries, e.g., during a myocardial infarct or stroke. This mechanism is disadvantageous, as CRP levels have been established to correlate with prognosis in these indications. In addition, it has recently been shown that CRP can directly affect blood pressure in rabbits [10]. It seems to justify the concept of essential hypertension as a complex immune-inflammatory disorder [11].

In patients with atherosclerosis (and vascular inflammation generally), levels of CRP usually are very low; therefore, hs-CRP tests are used to quantify its concentration. These high-sensitivity tests help to quantify a low degree of systemic inflammation in the absence of overt systemic inflammatory or immune disorders. hs-CRP tests have been standardized on several commercial platforms and can be accurately measured in fresh or frozen plasma [12].

Based on the obtained results, patients can be assigned to a risk group considering the development of the CVD: low risk (<1.0 mg/L), intermediate risk (1.0–3.0 mg/L), and high risk (>3.0 mg/L) [13]. However, the Multiethnic Study of Atherosclerosis (MESA) confirmed that women have higher median CRP levels than men. This gender difference indicates the need for establishing different cutoff values for cardiovascular risk for men and women [14]. In addition, CRP concentration was higher in smokers than in non-smokers and diabetic patients vs. non-diabetic. In contrast, CRP concentration was lower in alcohol drinkers than in non-drinkers and physically active people than in those not physically active [15].

In the large JUPITER study, hs-CRP levels were measured in populations from different continents. The median hs-CRP levels in the untreated population over four years of follow-up showed little change in these years, falling from 3.8 mg/L at baseline to 3.4 mg/L after four years. Thus, these results showed a strong persistence of hs-CRP concentrations, even in a selected population of people with high baseline values [16].

Many studies confirmed the predictive value of CRP as a cardiovascular risk factor. In a large individual participant meta-analysis of 160,309 people without a history of vascular disease from 54 long-term prospective studies, CRP was proven to have a similar importance as traditional cardiovascular risk factors and other markers of inflammation and showed nearly log-linearly with the risk of ischemic vascular disease and non-vascular mortality [15]. Akkoca et al. reported that high plasma hs-CRP level is a risk factor for peripheral arterial disease in cardiovascular adverse events development [17]. Su et al. conducted a study on the Chinese population and revealed that the hs-CRP level is related to subclinical carotid atherosclerosis, both in prediabetic patients and those with normal glucose metabolic status. A stronger relationship occurred in prediabetic patients; however, such a relationship was not revealed among patients with type 2 diabetes mellitus (T2DM) [18]. In the study by Andreas Pfützner et al., increasing hs-CRP levels were associated with an increased risk of developing T2DM in patients with all levels of metabolic syndrome. The haemoglobin A1c significantly correlated with hs-CRP levels and cardiovascular risk prediction in type 1 diabetes mellitus (T1DM) and T2DM. In addition, hs-CRP levels increased with β cells dysfunction and insulin resistance. Aspirin, statins, cyclooxygenase-2 inhibitors, and fibrates have found to lower hs-CRP levels in studied groups [19].

Interestingly, Aryan et al. found that adding hs-CRP to the list of risk factors for vascular events in patients with T2DM improves its predictive value of coronary heart disease events (CHDE) and microvascular complications. They also noted that the improvement of CHDE prediction achieved by implementing hs-CRP was more significant in patients with T2DM than in the general population [20]. Israr et al. reported that CRP, sialic acid, and low HDL-C are predictors of atherosclerosis, and they found a statistically significant correlation between CRP, sialic acid, and HDL levels [21]. Some studies suggest that hs-CRP should be used in combination with other biomarkers. Möhlenkamp et al. reported that if hs-CRP or coronary artery calcification (CAC) score are added to the Framingham Risk Score (FRS), they improve risk prediction in the general population [22]. They noted that when the CAC score is added to the combination of FRS and hs-CRP, it further enhances discrimination of coronary risk. An improvement in coronary risk prediction and discrimination was predominantly driven by CAC, whereas hs-CRP appears to have a role, especially in very low CAC scores. Overall, elevated CAC and hs-CRP levels were not only indicative of advanced coronary atherosclerosis and systemic inflammation, but in their presence, comorbidities appeared to be associated with a higher risk of death. Additionally, Koenig et al. reported that the combination of CRP and FRS leads to a more accurate prediction of a first coronary event than the results achieved by using those markers separately [23]. According to Diederichsen et al., hs-CRP also improves continuous net reclassification when applied to a model including traditional risk factors and CAC scores, but this improvement is not significant enough to justify adding CRP assessment to the list of traditional risk factors [24]. hs-CRP levels are known to be influenced by many factors, some of them being tissue damage, obesity, hypertension, smoking, diabetes mellitus, and even depression [13,25,26,27,28].

The American Heart Association (AHA) suggested that when evaluating CVD risk, levels of hs-CRP above 10 mg/L should be discarded, as they are probably the reflection of acute inflammation. In that case, it is recommended to repeat the test after two weeks to validate the results. However, Shrivastava et al. claimed that patients with hs-CRP levels above 20 mg/L are, in fact, at the very highest risk [27].

As an alternative to hs-CRP, Bayer wide-range CRP (wr-CRP) might be used to evaluate low-grade inflammation-associated cardiovascular risk. It has been confirmed that there is a strong correlation between wr-CRP and hs-CRP (for values below 5 mg/L). It is very promising, taking into consideration significantly lower costs of Bayer wr-CRP assay. However, further research is still needed because, despite good linearity, wr-CRP results were substantially lower than those of hs-CRP. Therefore, proper adjustments in the cutoff of the intermediate risk would be required [28]. Ziv-Baran et al. suggested changing the cutoff values between low and moderate risk groups from 1 to 0.9 mg/L, as it helped them eliminate differences in group classifications [29]. Recent studies confirmed that the CRP level can be lowered by leisure-time physical activity (LTPA) and that the frequency of this activity across adulthood is related to its anti-inflammatory effects [30]. Płócinniczak et al. showed that the hs-CRP concentration adds reliable information to the other comparable risk factors and risk scales among independent community-living elderly persons [31].

The latest findings by Quispe et al. based on the ARIC (Atherosclerosis Risk in Communities) participants suggested that inflammation was independently associated with atherosclerotic CVD regardless of atherogenic lipid levels and pooled cohort equation risk score among individuals without known CVD [32]. They found that participants with higher hs-CRP levels also had an independently increased risk of incidental heart failure and all-cause death than those with lower hs-CRP levels. Researchers noted that hsCRP levels should be regularly considered together with the measurement of lipids profile in a holistic and personalized approach to cardiovascular risk assessment and risk-based primary prevention. Elevated hs-CRP levels may serve as a risk enhancer irrespective of the baseline absolute risk determined by various atherogenic lipid measures and the pooled risk assessment of the cohort equation. People with elevated hs-CRP may also benefit from an active anti-inflammatory lifestyle and possibly anti-inflammatory medications; however, this requires further prospective validation in clinical trials.

In the study by Liu et al., the combination of elevated hs-CRP and hypertension significantly increased the cardiovascular risk in patients with stable, newly diagnosed CAD, supporting that hs-CRP could be used as a marker for stratification in high-risk patients [33].

Data from acute myocardial infarction (AMI) studies indicate that CRP levels may reflect the severity of the myocardial injury, and high CRP levels are associated with a poorer prognosis. Dedobbeleer et al. found in their studies that there was an increasing association between the occurrence of heart failure and the size of the CRP peak. There was also an inverse linear relationship between the left ventricular ejection fraction and the CRP peak. In addition, the CRP peak was lower when statins were administered. The researchers concluded that CRP is an indicator of the severity of ST-segment elevation myocardial infarction (STEMI) and can be treated as an indicator of complications during hospitalization in patients with STEMI [34].

The Hisayama Study revealed that elevated serum hs-CRP levels are an independent risk factor for the development of atrial fibrillation (AF) in a general Japanese population [35]. In turn, Buljubasic et al. revealed in their recent study that inflammation might mediate the mutual association of arterial hypertension and overweight, suggesting myeloperoxidase (MPO) as an inflammatory biomarker for arterial hypertension and hs-CRP for overweight [36]. In a healthcare-based study by Carrero et al., hs-CRP was clinically elevated in most healthcare-managed patients with AMI. Moreover, hs-CRP was associated with subsequent risk of major adverse cardiovascular events and death, with linear associations for hs-CRP ranging between 1 and 5 mg/L and plateauing thereafter to a sustained increased risk [37].

With the recognition of the critical relationship between arterial endothelial damage, inflammation, and coronary atherosclerosis, estimating hs-CRP appears to be essential in assessing cardiac risk and is an important prognostic factor in (1) AMI, (2) stroke, (3) peripheral arterial disease, (4) hypertension, (5) atrial fibrillation, and in the course of complications after AMI, such as heart failure. In addition, hs-CRP can be used to determine the likelihood of recurrence of cardiac events in patients with stable CAD, AMI. Evaluation of hs-CRP is recommended in patients at moderate risk of coronary heart disease to determine the need for further assessment and treatment. Moreover, the evaluation of hs-CRP seems to be justified in diabetic, overweight and prediabetic patients. The critical problem with this biomarker appears to be related to the high inter-individual variability of hs-CRP concentrations and the influence of many factors that may interfere together and affect the interpretation of this promising biomarker’s plasma concentration.

#### 2.1.2. Interleukin 6

IL-6 is a pleiotropic cytokine synthesized by lymphocytes, activated macrophages, astrocytes, ischemic myocytes, and endothelial cells. It acts via a hexameric complex composed of IL-6, IL-6 receptor (IL-6R), and glycoprotein 130 (IL-6/IL-6R/gp130). IL-6 and its receptor system structure are essential because of the many activities that this cytokine can exert. This glycoprotein is present in picogram per milliliter (pg/mL) amounts in the serum in physiology. The physiological processes in which IL-6 is involved are diverse and include primarily: (1) aging; (2) menstruation; (3) spermatogenesis; (4) liver regeneration; (5) skin proliferation; (6) participation in brain development; (7) bone-strengthening; (8) role in hematopoiesis; (9) function in regulating metabolism; (10) role in postprandial glucose levels; (11) regulating appetite and body weight control; (12) taking part in immune modulation/host defense (acute phase reaction, B lymphocyte differentiation, T helper activation, and T regulatory lymphocyte inhibition); (13) playing the critical role in the balance Th17-Treg cells in gut-associated lymphoid tissue (GALT) [38].

IL-6 levels can rise in virtually any inflammation, even during exercise by releasing IL-6 from skeletal muscle, or even after multiple traumas (proportional trauma), reaching micrograms per milliliter (µg/mL) values under severe conditions such as septic shock. In pathology, IL-6 basal function focuses on initiating the acute-phase response after injury or trauma, leading to inflammation or infection to remove infectious agents [39]. IL-6 mediates pro-inflammatory effects through trans-signaling, while through classical signaling, it is responsible for anti-inflammatory and regenerative effects [40]. Although classical IL-6 signaling occurs through membrane-bound IL-6 receptors, trans-signaling IL-6 is driven by systemic and localized increases in extracellular soluble IL-6 receptor (sIL6R) generated by proteolytic cleavage receptor “shedding” from the cell surface. sIL-6R can be activated by IL-6 and activate IL-6 signaling cascades via a constitutively expressed gp130 coreceptor. Thus, IL-6 trans-signaling enables the activation of IL-6 signaling pathways in cells that do not express IL-6R.

IL-6 signaling is involved in CAD and has recently become a focus of attention due to the global COVID-19 pandemic. This pro-atherogenic cytokine reached elevated serum levels during the cytokine storm generated by SARS-CoV-2 and was also associated with smoking or classical cardiovascular risk factors that promote inflammation and obesity. IL-6 levels were found to be associated with dyslipidemia, hypertension, and glucose dysregulation and were associated with poor outcomes in patients with unstable angina or AMI [40].

After cessation of the stress factors, IL-6 synthesis should finish. However, when it does not, uncontrolled, excessive, or persistent IL-6 production plays an essential role in the development of various pathologies such as inflammatory diseases and cancers, indicating that IL-6, although necessary, can also be dangerous for the patient. To sum up, the proper IL-6 expression is essential for host defense. It is strictly controlled by various intracellular mechanisms, whose dysregulation may cause abnormal IL-6 expression leading to multiple acute systemic and chronic inflammatory diseases [41].

It is now well recognized that atherosclerosis is a chronic inflammatory disease occurring in response to vascular injuries that results from accumulating lipids within the walls of arteries. Chronic inflammation in CVD appears to be associated with the oxidative/anti-oxidative homeostasis controlled, among others, by IL-6. Probably, reducing vascular inflammation itself could lower the rates of critical cardiovascular events.

Plasma levels of inflammatory biomarkers such as IL-6 are known to predict future cardiovascular events. IL-6 is a presumable candidate biomarker for predicting cardiovascular risk because of its role in inducing CRP synthesis in the liver and its classification as an upstream cytokine to reflect inflammation. IL-6 is abundantly released in the process of atherosclerosis development. There is strong evidence indicating that among those with elevated hs-CRP, IL-6 is associated with cardiovascular risk in addition to traditional risk factors. What is more, there is an established connection between IL-6 levels and endothelial dysfunction and subclinical atherosclerosis, and there are reports from multiple studies indicating that IL-6 signaling plays a role in atherothrombosis [42]. It has also been shown that increased IL-6 plasma level is linked to increased risk of AMI in healthy men. During the acute phase of AMI, there is an augmentation in IL-6 and IL-6 receptor levels, which is probably related to plaque instability [43].

In a study by Subirana et al., researchers determined the predictive capacity of CAD for selected biomarkers from various pathways: (1) inflammation, such as TNF-α, interleukin 10 (IL-10), IL-6, monocyte chemoattractant protein-1 (MCP-1), and CRP; (2) oxidation (glutathione peroxidase 1 (GHS-Px)) and (3) metabolism (adiponectin, leptin, and insulin) regardless of classical risk factors. After adjusting the results for age and gender, only IL-6, insulin, and TNF-α were correlated with CAD incidence. However, in the fully corrected model, it was found that there was a significant independent effect for CAD incidence only for insulin and TNF-α was maintained [44].

Due to its presumed essential role in the pathogenesis of arteriosclerosis, IL-6 is a primary target in research for effective medication. Generally speaking, treatments aiming to lower systemic inflammatory markers have a beneficial effect on complications caused by atherosclerosis. Yet, there is an uncertainty in those findings, because the reduction in inflammatory markers is frequently linked to the mitigation of “traditional” risk factors such as cholesterol levels, which are well-known to have an essential role in arteriosclerotic plaque formation. Tocilizumab is a monoclonal humanized antibody acting by blocking soluble and membrane-bound IL-6R, which results in clear improvements of endothelial functions, a significant reduction of CRP concentrations, and systemic inflammation parameters. It is currently a treatment for rheumatoid arthritis but is being investigated to be used as an anti-arteriosclerotic therapy due to its anti-inflammatory properties. Still, tocilizumab worsens the atherogenic lipid profile, a “traditional” arteriosclerosis risk factor, because it increased total cholesterol (TC), LDL-C, and triglycerides [45]. It is challenging to evaluate if the tocilizumab positive, anti-inflammatory effect outruns the possible adverse effects of changing the patient’s lipid profile. For now, the possibility of treating arteriosclerosis with tocilizumab stays a distant possibility due to its uncertain cardiovascular outcomes. The targeting of IL-6 on treatment development (not only) in cardiovascular disease and COVID-19 is still under ongoing research, although tocilizumab has been shown to be effective in reducing the pro-atherogenic effects of IL-6, and it has been suggested that it improves patient survival with COVID-19 [40].

Recent genetic polymorphism studies suggest that IL-6 receptor signaling seems to have a causal effect on CAD. Two groups of researchers studied the population distribution and impact of Asp358Ala polymorphism in the IL-6R gene, which is a variant that reduces IL-6 signaling, which causes a significant drop in systemic inflammation. One of the groups showed that every inherited copy of 358Ala inherited decreases the CAD risk by 3.4% (95% CI, 1.8–5.0) (51,441 patients with CAD and 136,226 controls) [46]. The second study involved 25,458 CAD patients and 100,740 controls, and their results showed a reduction of 5% (95% CI, 3–7) [47]. The results of these studies open an innovative route for the use of IL-6R blockade to prevent CAD in the future. Still, the only currently available IL-6 blocker is tocilizumab, which is described above. Even if it has a similar pattern of blocking the IL-6R pathway to the 358Ala polymorphism, it also has the disadvantageous effect of changing the patient’s lipid profile. Using it as an anti-arteriosclerotic drug requires further investigations with longer durations [48].

What is interesting, while multiple studies show that lower IL-6 concentration is correlated with lower cardiovascular risks, it has been reported that decreased levels of inflammatory biomarkers, including hs-CRP and IL-6, were associated with worse health-related quality of life (HRQoL) in patients with CAD and heart failure [49].

In SOLID-TIMI 52 Trial, Fanole et al. determined that IL-6 is associated with an increased risk of severe adverse cardiovascular events, including heart failure. This relationship was independent of traditional clinical risk predictors and markers, including hs-CRP, Lp-PLA2 activity, highly sensitive troponin I (hsTnI), and B-type natriuretic peptide (BNP). These findings support the concept of IL-6 as a potential therapeutic target in patients with unstable ischemic heart disease. IL-6 was also associated with plaque instability in acute coronary syndrome (ACS). However, its prognostic significance here is still debatable. The researchers concluded that IL-6 might be helpful in risk stratification in patients after an acute coronary syndrome. Their findings support studies investigating a possible role for interleukin-6 as a therapeutic target in patients with unstable CAD [50].

An excellent summary of the importance of IL-6 as a potential biomarker of cardiovascular risk is the meta-analysis by Zhang et al. [51]. It was based on 5400 CVD cases and 14,607 matched non-CVD controls out of 288,738 healthy subjects. Researchers have revealed that significantly higher levels of unhealthy people with raised IL-6 were associated with the risk of cardiovascular events over the 7-year average. Thus, they showed that inflammatory pathways act chronically, causing symptomatic cardiovascular disease. Hence, prophylactic strategies should also consider pro-inflammatory cytokine profiles, especially IL-6 analyzed here, in selected individuals. It was found that IL-6 levels were positively associated with hypertension and hypercholesterolaemia but negatively associated with triglyceride levels [51]. However, the researchers noted that the interactions of IL-6 with lipid metabolism are quite complex. Specific polymorphisms in the promoter region of the IL-6 gene have been shown to result in higher levels of circulating IL-6, which are associated with higher levels of circulating triglycerides but not total cholesterol levels [52], suggesting that differential expression of the IL-6 gene may affect the lipid profile.

One can conclude that although IL-6 turns out to be a good marker in many cases, due to the multifaceted nature of the processes participating and the polymorphisms of IL-6 and IL-6R genes, it is not ideal for all of the studied persons.

#### 2.1.3. Lipoprotein-Associated Phospholipase A2

Lipoprotein-associated phospholipase A2 (Lp-PLA2) is a calcium-independent lipase that hydrolyzes the acetyl group of platelet-activating factor (PAF) as well as oxidizes phospholipids in LDL [53]. Compared with other members of the phospholipase A2 superfamily, it can catalyze hydrolysis at the sn-2 position of phospholipids [54,55,56,57].

Plasma levels of Lp-PLA2 have been identified as a biomarker of vascular inflammation and atherosclerotic vulnerability, which help predict future cardiovascular events [58]. The enzyme is mainly produced by monocytes and macrophages and circulates in plasma, being associated with LDL and HDL. The Lp-PLA2 expression is activated by apolipoprotein CIII (apo CIII, a protein that is found in triglyceride-rich lipoproteins such as chylomicrons, very-low-density lipoprotein (VLDL), and remnant cholesterol, whose function is inhibition of lipoprotein lipase and hepatic lipase), oxidized LDL (oxLDL, lipoproteins formed under the influence of reactive oxygen species, which are not recognized by receptors for primary LDL), serum amyloid A and leukocytes. In contrast, nitro-oleic acid downregulates Lp-PLA2 expression [59].

Lp-PLA2 has a dual role in the inflammatory process, depending on the type of lipoprotein with which the enzyme is associated [60]. HDL-Lp-PLA2 has an anti-inflammatory, anti-oxidative, and anti-atherogenic role, while the LDL-Lp-PLA2 expresses pro-inflammatory and pro-atherogenic effects. It should be underlined here that Lp-PLA2 is carried bound mainly to LDL in the circulation. The hydrolysis of oxLDL lead to the release of lysophosphatidylcholine (lyso-PC) and oxidized free fatty acids (OxFFA), which are the triggers of inflammatory cascade by induction of chemotaxis of monocytes and leukocytes and promotion of their entry in the sub-intimal space of the artery wall [56]. What is more, these substrates attach to activated macrophages through scavenger receptors and are phagocytized, leading to foam cells’ formation [55]. These cells play an essential role in atherosclerosis. They induce the accumulation of lipids, which lead to fatty streak formation in the vascular wall [57]. The muscle cells also migrate to the intima, where these cells start to produce collagen and elastin, which are involved in stabilizing the atherosclerotic plaque [55]. Lyso-PC is also engaged in the production of reactive oxygen species (ROS). When lyso-PC activates the endothelial nicotinamide adenine dinucleotide phosphate (NADP), it oxidizes and induces the endothelial nitric oxide synthase (eNOS). Those pro-inflammatory and pro-oxidative effects of Lp-PLA2 are involved in the pathogenesis of atherosclerosis [56].

On the other hand, enzyme activity associated with HDL can play the opposite role. Studies have shown that HDL-Lp-PLA2 decreases endothelial adhesiveness and macrophage recruitment to prone lesion sites. It is confirmed by the dual action of Lp-PLA2, which depends on the lipoprotein to which the enzyme is associated. Lipase associated with HDL shows an anti-atherogenic role, while LDL-Lp-PLA2 stimulates the process of atherosclerosis [55]. Lipase can also be associated with Lp(a), and this complex may play a role similar to that observed for the LDL-Lp-PLA2 in the artery wall. HDL-Lp-PLA2 level testing has shown that it is reduced in patients with combined hyperlipidemia, primary hypertriglyceridemia, pre-diabetes, and metabolic syndrome, while LDL-Lp-PLA2 level is elevated in these patients [60]. What is more, research has shown that Lp-PLA2 was increased in the subjects with the incidence of CVD; also, the patients with heart failure have an elevation of Lp-PLA2 levels [55]. This is why Lp-PLA2 is one of the most promising atherosclerosis biomarkers that can be useful in assessing cardiovascular risk in asymptomatic patients [56]. That inflammatory biomarker was also approved by the United States Food and Drugs Administration (FAD) as a predictor of ischemic stroke. Lp-PLA2 is considered to be a more specific marker of cardiovascular risk. However, many epidemiological studies have found inconsistent results regarding whether this lipase can be used to predict atherosclerosis. That is why it may be helpful to use a combination of hs-CRP and Lp-PLA2 in the prediction of the risk of CVD, including CAD and stroke [61].

Since we know that Lp-PLA2 is involved in the atherosclerosis process, in particular, when it is associated with LDL, scientists focused on finding a therapeutic strategy to reduce the Lp-PLA2 plasma level. One of these strategies may be decreasing LDL cholesterol levels, and the result of that action could be a reduction in activity Lp-PLA2. Several cholesterol-lowering treatments, such as statins, ezetimibe, and omega-3 fatty acids, were found to reduce Lp-PLA2 activity [56].

Several epidemiological studies in the general population have revealed a correlation between Lp-PLA2 levels and traditional cardiovascular risk factors [62,63,64,65]. Particularly significant are the results of analyzes conducted by The Lp-PLA2 Studies Collaboration on a group of 79,036 participants from 32 prospective studies (yielding 17,722 incident fatal or non-fatal outcomes during 474,976 person-years at risk). On their basis, it has been shown that the activity and mass of Lp-PLA2 show a continuous relationship with the risk of coronary heart disease, which is similar in magnitude to that of non-HDL cholesterol or systolic blood pressure in the studied population. Moreover, the relationships of Lp-PLA2 activity and mass are not exclusive to vascular performance, and vascular compounds are at least partly lipid dependent [62]. However, these researchers pointed to the need for more profound studies of extravascular outcomes, mainly since the recorded association of Lp-PLA2 with the risk of non-neoplastic and non-vascular deaths could be attributed, at least in part, to comorbidity at the start of the study. Additionally, the researchers noted a potential limitation of any observational studies of circulating Lp-PLA2 is that the enzyme in the blood may be an imperfect indicator of its importance in atherosclerotic plaque. In addition, the existence of mutations that cause loss of function in the PLA2G7 gene, which are common in East Asian populations, have been shown to effectively abolish Lp-PLA2 activity (or, in heterozygotes, significantly reduce the activity). Interestingly, the vascular risk is not lower in people with such mutations. On the other hand, the known genotypes related to Lp-PLA2, which are common in people of continental European descent, have only a weak effect on Lp-PLA2 activity. The researchers suggested that randomized studies of potent reversible pharmacological inhibitors of Lp-PLA2 activity are needed to assess whether the modification of Lp-PLA2 can reverse vascular risk unequivocally.

Overall, Lp-PLA2 appears to be a promising biomarker as an indicator of the complex processes contributing to plaque formation, although more research is needed to evaluate its significance.

A summary of findings about diagnosis and prognosis from studies mentioned in Section 2.1 concerning recognized cardiovascular biomarkers can be found in Table 1.

### 2.2. Potentially New Biomarkers

#### 2.2.1. MicroRNA

MicroRNAs (also known as miRNAs or miRs) represent a group of about 17–25 nucleotides of long, non-coding RNAs that have been shown to modulate gene expression at the translational level by interfering with the 3’ untranslated region (UTR) of messenger RNA [66]. Circulating miRNAs are highly stable and considered to be novel biomarkers for the diagnosis and/or prognosis of CVD. Many studies concerning miRNA as a potential biomarker in atherosclerosis were published in the last few years, but new miRNAs are still emerging as possible clinical biomarkers in diagnosing atherosclerosis.

MiRNAs by interactions with mRNAs impact protein synthesis; hence, they play a significant role in developing numerous diseases. The effect of harmful stimuli and the influence of miRNA may be the cause of atherogenesis [67]. It has been found that miRNAs are regulated in different stages of atherosclerosis, from activation and proliferation to cellular senescence. For instance, miR-21 expression was increased in peripheral blood mononuclear cells (PBMCs) in patients with severe vascular disease and AMI in the medical history and continues rising along with the severity of atherosclerosis [68,69]. miR-21 high abundance was observed in macrophages [70] and was revealed to affect foam cell formation [71]. Berkan et al. found that the formation of atherosclerotic plaque was associated with miR-486-5p downregulation [72].

In the recent research for new miRNAs, conditions such as hypothyroidism, obstructive sleep disorder, stent implantation, hyperhomocysteinemia, and the antiphospholipid syndrome were considered as factors that could have influenced the results. In patients with hypothyroidism and raised TSH level, miR-146a emerged as a possible predictor of atherosclerosis and its severity [73]. It may be due to dysfunction in endothelial cells caused by raised TSH levels [74], which later promotes the proliferation of smooth muscle cells. Another study found that patients with obstructive sleep disorder and increased maximum carotid intima-media thickness (IMT) levels of miR-664a-3p were remarkably higher than in control groups [75]. Predicting adverse ischemic events may be improved by miR-195 in patients after stent implantation. miR-195 inhibits the proliferation of smooth muscle cells by modulating its phenotype; it also inhibits neointima. Thus, its decrease correlates with the increase of possible adverse ischemic events in two years after stenting [76]. Hyperhomocysteinemia increases the risk of atherosclerosis, probably causing the inflammatory response and later proliferation in vascular smooth muscle cells (VSMC) [77]. Moreover, in the study conducted by Liu et al., the upregulation of miR-217 in patients with hyperhomocysteinemia and concomitant atherosclerosis was remarkably higher than in control groups [78]. In addition, Menghini et al. revealed that by inhibiting the expression on silent information regulator 1 (a homeostasis controller of endothelial dysfunction), miR-217 triggers the senescence of endothelial cells [79].

In turn, miR-126 was shown to prevent atherosclerotic lesions from forming by inhibiting Notch1 inhibitor delta-like 1 homolog, and miR-143 was shown to repress smooth muscle cell proliferation [80]. Therefore, their downregulation may be a valuable predictor of cerebral atherosclerosis in patients. This study found a correlation between miR-126 and miR-143 and cerebral atherosclerosis and its severity [81]. In patients with antiphospholipid syndrome, the antiphospholipid antibodies were shown to modulate the expression of proteins and miRNAs, leading to atherosclerosis. This study found that in this group of patients, the ratio of miR-19b and miR-124, which take part in inflammation and thrombosis [82], might be a valuable biomarker of the early development of atherosclerosis [83]. Another study has revealed that the overexpression of miRNA-210 increases the stability of the fibrous cap of atherosclerotic carotid lesions in Apoe -/-mice by triggering smooth muscle cells’ proliferation and survival [84]. Moreover, the increased expression of miR-146a or miR-146b in patients with CAD was associated with an increased risk of atherosclerosis [85]. Among the biomarkers of the advanced atherosclerotic plaques in the coronary arteries, several miRNAs (miR-21, miR-92a, and miR-99a) have been found to be upregulated in CAD patients’ circulation. Interestingly, miR-21 levels differed between symptomatic and asymptomatic plaques, indicating that this miRNA could be a potential biomarker for distinguishing symptomatic plaque burden. According to this study, miR-221 and miR-222 were recognized as anti-atherosclerotic miRNAs and can be used as diagnostic, theranostic, and prognostic biomarkers in atherosclerosis and inflammatory diseases [86]. In other interesting meta-analyses based on thirteen studies related to miRNAs and CVD, involving 16,484 subjects, conducted by Liu et al., significant associations between microRNA-146a rs2910164 polymorphism and CVD risk were observed in the total population, as well as in subgroup analyses of ethnicity. Similarly, increased CVD risk for microRNA-196a2 rs11614913 and microRNA-499 rs3746444 was also disclosed [87].

Moreover, Condrat et al. concluded [88], based on the analyses of the studies focusing on AMI and miRNAs, that the same four miRNAs (miR-1 miR-133a, miR-208a/b, miR-499 classically referred to as the myomiR family) are upregulated and increase in circulation shortly after AMI: (1) miR-1 (identified in both cardiac and skeletal muscles, important for early cardiogenesis, promotes apoptosis and worsens oxidative stress in damaged cardiomyocytes; its release in AMI suggests necrotic death of cardiac myocytes as their source, downregulated in hypertrophic hearts); (2) miR-133a (plays a key role in promoting cardiogenesis, heart function, and pathology, together with miR-1 control the early cardiogenesis stage and mediates cardiac conductance and automaticity [89], is downregulated in the infarct area, in contrast to miRNA it inhibits apoptosis in damaged cardiomyocytes and adverse cardiac remodeling [89]); (3) miR-208a/b (miR-208a—encoded within the α-cardiac muscle myosin heavy chain genes and miR-208b—encoded within introns of the β-cardiac muscle myosin heavy chain genes, its overexpression promotes cardiac hypertrophy, both are important for the late cardiogenesis, control sarcomeric contractility, and express cardioprotective properties [89]); and (4) miR-499 (encoded, similarly to miR-208b within introns of the β-cardiac muscle myosin heavy chain genes, which is important for late cardiogenesis). More specifically, the analysis by Wang et al. showed that miR-208a has the highest accuracy in diagnosing AMI, with levels increased significantly as early as one hour after occlusion in 90% of AMI patients tested and 100% of AMI patients within four hours [90]. On the other hand, when Liu et al. compared samples from AMI patients by analyzing plasma levels of miR-1, miR-208, and miR-499; miR-499 has the highest predictive value and greater reliability than TnT and CKMB, which are traditional cardiac biomarkers [91]. In turn, Liu et al. [92] discovered that miR-208a and miR-370 are promising diagnostic biomarkers for discriminating CAD and may facilitate patient care management. They also found that the combination of the two miRNAs may be more productive than either miRNA alone to diagnose CAD.

Additionally, Chen et al. [93] disclosed, based on the selected clinical and basic data, that eight miRNAs (miR1, miR-21, miR-126, miR-133, miR-145, miR-208, miR-223, and miR-499) have shown significant clinical value as biomarkers for CADs. Several of them have been identified in other studies, but additional ones such as miR-21, miR-126, miR-145, and miR-223 deserve an explanation. miR-21 can regulate cardiac fibrosis. Circulating miR-21 levels were significantly elevated in patients with coronary artery stenosis in response to 24 h cardiac stress after dobutamine stress echocardiography [93]. In comparison, circulating miR-126 levels were decreased in patients with AMI after the onset of symptoms compared with the healthy group and were negatively associated with hsCRP. It is interesting that the administration of aspirin was found to reduce circulating miR-126 levels in human [94]. Circulating miR-145 levels were significantly decreased in CAD patients compared with non-CAD subjects, and the reduced levels of miR-145 were associated with the severity of CAD [95]. In turn, circulating miR-208 levels were increased in CAD and were associated with the mortality of CADs; thus, the researchers concluded that miR-208b could be one of the mortality-predicting biomarkers for AMI after adjustment for age and sex [96]. Circulating miR-223 levels, contrary to many other miRNAs, were higher in CAD patients and associated with increased mortality and an increased risk for future AMI; they were disclosed to be a promising prognostic biomarker of predicting cardiovascular death [97].

Despite significant advances in miRNA research focusing on the diagnosis and treatment of CVD, many questions remain open regarding the regulation of gene expression based on miRNAs. The complex nature of miRNA regulation leads to many of the challenges researchers face. Moreover, the identification and validation of these miRNA targets, particularly in pathological conditions, is challenging due to the heterogeneity of miRNA regulatory mechanisms in different cell types. Despite these challenges, there is a great need to study the variability in miRNA-based phenotype regulation in CVD. The interactions between multiple miRNAs, with their shared cognate mRNAs, which show significant effects on gene regulation in various states, should be further considered. Moreover, the continuous discovery of entirely new mechanisms regulating CVD development, including circulating or tissue-resident miRNAs, offers new hope for innovative diagnostics and treatment [97]. On the other hand, the effect of comorbidities on the circulating miRNAs levels is largely unknown. In addition, drugs such as statins, anticoagulants, and antiplatelet drugs can affect the circulating miRNA levels [97]. Many miRNAs in the cardiovascular system that target different proteins and mRNAs have been studied and selected; the most promising ones have been presented in this chapter.

#### 2.2.2. Osteocalcin

Osteocalcin (OC), also known as bone glutamic acid protein (BGLAP), is a non-collagenous synthetic protein secreted mainly by osteoblasts. OC regulates the bone extracellular matrix by binding to calcium ions and hydroxyapatite crystals; thus, it is considered a traditionally bone formation marker [98,99].

The maturation of OC is quite complicated and not fully understood. OC is initially synthesized as a proprotein by osteoblasts, chondrocytes, and osteoblast-like VSMCs. Next, OC proprotein experiences signal peptide removal and is converted into an uncarboxylated isoform (uOC). The active form of vitamin D (1,25(OH)_2_D_3_) increases the OC expression in humans and rats, as opposed to mice, where it decreases OC expression [100]. Animal experiments have demonstrated that only the uOC isoform exhibits hormonal activity; however, data from clinical observational trials are conflicting [101]. Then, uOC, thanks to the γ-glutamyl carboxylase (GGCX) action and its coenzyme—vitamin K, is converted into carboxylated OC (cOC). About 20% of cOC enters the bloodstream, and the rest enters the bone and binds to calcium deposits in the bone matrix. cOC inhibits the bone resorption activity of osteoclasts. During active bone resorption, cOC may be transformed into uOC and an undercarboxylated OC isoform (ucOC) again following decarboxylation. cOC and uOC may enter the blood circulation. The ucOC cannot be released into the blood in the vitamin K environment due to its reduced binding affinity to bone minerals [101]. The mechanism of cOC entry into the blood circulation and whether it promotes or inhibits the calcification of blood vessels is not currently precise [100]. The ucOC transference into cOC could be enhanced by vitamin K [102], and the level of both OC isoforms could be dependent on diet [103]. Some studies exposed that cOC and ucOC were linked with energy metabolism and atherosclerosis [104]. Low ucOC was associated with abdominal aortic calcification in the male cohort [105,106].

OC has several features of the hormone and has recently been linked to increasing extra-bone biological roles. These extra-bone functions include the following: (1) influencing brain development and function, which sheds new light on the cause of cognitive decline with age [107]; (2) stimulating the expression of cyclin D1 and insulin in pancreatic β cells and adiponectin (an insulin-sensitizing adipokine) in adipocytes and improving glucose tolerance [108]; (3) linking the pathway between central obesity and insulin resistance [109]; and (4) promoting male fertility by increasing testosterone production [110,111].

One of the potential threads of inquiry is OC interaction with the vascular system and its supposed role in vascular calcification or atherosclerosis. Recent studies have evidenced that OC is closely related to engaging in atherosclerotic changes. Therefore, it may be correlative as a specific arteriosclerosis marker in the future [112].

OC was found to influence vascular calcification, which is a risk factor for mortality and morbidity and an independent risk factor for cardiovascular disease. Vascular calcification is now considered an active, cell-mediated, and complex regulated process, and OC plays an essential function. It should be noted that in bone, biomineralization occurs through endochondral or membranous ossification programmed by chondrocytes and osteoblasts. Interestingly, VSMC can transform into osteoblast-like cells showing osteogenic fingerprints characterized by a decrease in smooth muscle cell markers and an increase in osteogenic markers, such as alkaline phosphatase, or the OC just presented. This active osteogenic process can be triggered by oxidative stress [112]. Rached et al., in their study, identify FoxO1 as a crucial regulator of osteoblast physiology and provide a direct mechanistic link between oxidative stress, atherosclerosis, and bone remodeling regulation [113].

The development of arteriosclerosis is related to endothelial apoptosis and, thus, the dysfunction of the endothelium [114]. Additionally, a high level of free fatty acid (FFA) damages endothelial function [115]. OC stimulates the phosphatidylinositol 3-kinase (PI3-kinase)/Akt signaling pathway and inhibits endothelial cell apoptosis induced by FFA [116]. On the other hand, it is also known that OC encourages endothelial progenitor cells (EPCs), which stimulate the proliferation and formation of vascular endothelium in patients with coronary atherosclerosis [117]. The study by Flammer et al. suggested that EPCs can improve atherosclerotic changes and seem to be an appropriate marker to distinguish clinically stable and unstable atherosclerotic patients [118]. Decreased EPCs levels were observed in diabetic patients, and it has been shown that peripheral vascular disease is associated with an extensively low number of EPCs. This decrease in the EPCs level may be involved in the pathogenesis of peripheral vascular complications observed in diabetic patients [119].

As was previously mentioned, recent studies revealed that OC, besides bone remodeling, is also involved in glucose and lipid metabolism (ucOC can act directly on pancreatic beta cells and adipocytes, regulating insulin secretion and insulin sensitivity) [120]. Insulin has an impact on the increase of OC synthesis. On the other hand, OC encourages insulin production and secretion from pancreatic beta cells; therefore, it is considered to enhance insulin sensitivity and glucose tolerance [121,122]. A lower circulating OC level was found to be related to the future development of T2DM independent of conventional risk factors in Japanese postmenopausal women [121]. In addition, decreased OC was associated with the presence of atherosclerotic plaques and correlated with CRP in a cohort of adult and T2DM patients. Other studies showed that low OC levels accompanied carotid atherosclerosis in T2DM patients [123] and Chinese postmenopausal women [124]. Weakened OC was associated with a disorder of the carotid arteries in patients compared to the healthy group, with half of the patients having diabetes [125]. Moreover, in the Changfeng Study, based on 1077 male participants, it has been disclosed that OC is independently associated with carotid atherosclerosis in male individuals with normal glucose tolerance and that the OC level may be implicated in glucose metabolism and atherosclerosis [126]. Interestingly, the prevalence of carotid plaque significantly decreased with increasing OC level after adjusting for traditional CVD risk factors in euglycemic middle-aged and elderly male adults [126].

In addition to diabetic patients, patients with chronic kidney disease (CKD), especially end-stage renal disease, are another important group in OC research. They are susceptible to CVD and mineral bone disorders (CKD-MBD—CKD–mineral and bone disorder) [2]. Since the vitamin K-mediated carboxylation pathway is crucial for the development of c-OC, and CKD patients often exhibit subclinical vitamin K deficiency, so they may reveal the OC changes. OC levels are known to increase in hemodialyzed (HD) patients in both male and female patients than in their respective controls [127]. Dephosphorylated, undercarboxylated OC (dp-ucOC) levels increased as CKD worsened [128]. Jia et al. showed that the serum OC level is associated with abnormal CKD-MBD mineral parameters; in this study, OC proved to be a risk factor for vascular calcification. However, in this study, OC was not classified as cOC and unOC. Whether unOC is more directly related to vascular calcification requires further research [129]. Compared to healthy subjects, HD patients presented significantly reduced serum levels of c-OC and increased serum levels of unOC [129]. In another study that enrolled HD patients (*n* = 189) and pre-dialysis CKD patients (*n* = 89), the serum ucOC/intact OC ratio > 1.0 was observed in about 71.4% of HD patients, especially those with high bone turnover [130] Another study by Lin et al. [131] revealed that higher serum OC level was associated with lower vascular reactivity index (VRI) and poorer endothelial dysfunction among kidney transplant recipients [131,132]. Impaired vascular reactivity was found to be directly associated with an increased mortality rate in end-stage renal disease [131].

In The Health In Men Study conducted by Yeap et al. that involved 3542 community-dwelling men aged 70–89 years, the level of total serum OC was predictive of all-cause and CVD-related mortality in a U-shaped distribution, whereby increased mortality rates were associated with either higher or lower levels of OC [133]. The results were contrary to those of the study by Hwang et al. [134]. In this study, based on 1290 men aged 40–78 regularly followed at the Health Promotion Center on an outpatient basis and during hospitalization for a mean of 8.7 years, the serum total OC level was not associated with the development of CVD after adjusting for other CVD risk factors [134]. As the researchers noted, because of these inaccuracies, further research is needed to clarify whether the underlying mechanisms involve altered bone turnover or whether they specifically relate to the biological activity of OC [134].

Hence, although recent studies suggest that serum OC is associated with cardiovascular events, it is still debatable whether there is an independent association between OC and atherosclerotic CVD. Many issues remain to be clarified. First, it should be emphasized that total OC may not be a valuable measure of the risk of vascular calcification and atherosclerosis, but which isoform of OC is biologically active should be clarified, as the data are contradictory. Second, difficulties arose in measuring ucOC and cOC, as there are few tests. It is unclear which test system provides the most accurate measurements due to comparability and heterogeneity issues for OC [129]. It should be noted that some studies have highlighted the observed differences between the various ethnic groups [135]. Third, OC shows a circadian rhythm with a night peak, and therefore, the time of blood sampling may also contribute to the variability of the obtained results [136]. Fourth, we are dealing with the influence of vitamin K [137] and vitamin D and the importance of supplementing these vitamins and bone metabolism status generally on the level of OC, which should also be taken into account. All these factors influence the interpretation of the results and should be reconsidered before OC becomes a recognized cardiovascular biomarker.

In conclusion, according to current knowledge, the level of OC seems to be associated with atherosclerosis and vascular calcification. However, whether OC can become a helpful tool for predicting the future risk of atherosclerosis, the question still seems open. Serious difficulties are associated with the presence of OC in different isoforms and variability of OC in diverse populations, which makes it difficult to conclude and compare between each other.

#### 2.2.3. Angiogenin

Angiogenin (ANG) is a 14 kDa molecular weight extracellular protein, a member of the ribonuclease (RNase) superfamily of enzymes (also known as RNase 5); it was initially identified in a medium conditioned with tumor cells. ANG was detected in human tissues and fluids (plasma, amniotic, tumor microenvironment, and cerebrospinal fluid). It was found localized to different cellular compartments under different conditions, such as growth (nuclear) and stress (cytoplasmic). Due to ANG properties, is involved in many processes, such as (1) tumorigenesis; (2) neuroprotection; (3) inflammation; (4) innate immunity; (5) reproduction; and (6) regeneration of damaged tissues [138].

ANG, one of the strongest angiogenic factors, interacts with endothelial cells and induces a cellular response, initiating the process of new blood vessel formation [138].

Atherosclerosis is a complex process [139,140,141], which was lately linked to angiogenesis [142,143]; some studies show the importance of ANG, mainly in the development of microvessels inside the core of the atherosclerotic plaque. These vessels are thin-walled and lined with a discontinuous endothelium without supporting the VSMCs, making the vessel wall very sensitive and prone to cracking. Hemorrhage within the plaque destabilizes it, leading to occlusive thrombosis and clinical symptoms of ACS. ANG is required for vascular endothelial factor (VEGF) to stimulate angiogenesis [144]. In addition, it plays a vital role in the interaction of proteases that activate wound healing, such as the metalloproteinase family, and in the stimulation of tissue plasminogen activator (tPA) to produce plasmin [145,146]. These proteases are also associated with the destabilization of atherosclerotic plaque. Hence, high levels of angiogenic factors could be potential markers of unstable plaque in ACS and could be a risk marker of future ACS [144].

Kręciki et al. evaluated the relationship between plasma ANG levels, biochemical risk factors, and three-vessel CAD. The study included 107 patients with three-vessel CAD and 15 controls. The control group showed ACS, a positive exercise stress test, and abnormal segmental contractility, but they did not show coronary stenosis in their angiograms. In both studied groups, ANG, resistin, adiponectin, IL-8, and TNF-α were measured. The severity of CAD was expressed as Gensini scores (a widely used method of quantifying angiographic atherosclerosis, where zero score means no atherosclerotic disease). There was a significant difference in ANG concentration between patients with CAD and the control group (414 ng/mL vs. 275 ng/mL, *p* = 0.02). Moreover, ANG was increased in patients with higher Gensini scores (*p* = 0.06) [147].

In turn, Tello-Montoliu et al. measured ANG levels in ACS [144]. The study followed 396 patients (63.4% males, mean age 67 years) diagnosed with ACS who were compared with 44 patients with stable CAD as well as 76 healthy control group (Gensini score-*p* < 0.001). Clinical follow-up was six months, in which some patients developed cardiovascular death, recurrent ACS, revascularization, and heart failure [144]. ACS patients revealed significantly elevated plasma angiogenin levels compared with the healthy control group and patients with stable CAD. The complete profile of the ACS group was as follows: (1) high troponin T level, (2) electrocardiographic changes, and (3) raised ANG, which all were independently associated with more adverse events at six months follow-up (*p* = 0.008) [144].

Jiang et al. studied ANG as a novel biomarker in heart failure. They showed that ANG levels were positively correlated with N-terminal pro-B-type natriuretic peptide—(NT-proBNP) (*p* < 0.001). High ANG levels (≥426 ng/mL) were found to be an independent predicting factor of all-causes death of heart failure with preserved ejection fraction (HFPEF). Levels of ANG were significantly increased, ranging between 290 and 450 µg/min, among patients with advanced outcomes (high troponin T level) [148]. Other researchers, Patel et al. [149], performed their study on a group of 109 patients with congestive heart failure (CHF) (85 males, mean age 60 years) and 112 control patients with normal cardiac function. They measured BNP and ANG levels. Their results showed that ANG levels were significantly higher in CHF (*p* < 0.001) compared to the control group. ANG was found to be positively correlated with age, plasma glucose, insulin, and BNP (all *p* < 0.001) level and negatively associated with diastolic blood pressure (*p* = 0.04). Angiogenin was a modest factor in the presence of CHF (*p* < 0.001). This study showed that high ANG levels were predictive of adverse events such as death during follow-up.

ANG is an unusual molecule for its proposed functions in RNA metabolism. It is worth noting that many biological processes are related to ANG functions, such as angiogenesis and neovascularization, stress adaptation and survival, cell signaling, and the maintenance of stem cell homeostasis. Given the established roles of ANG in the etiology of disorders such as cancer, infection, and rare diseases, such as amyotrophic lateral sclerosis, further investigations of the molecular mechanisms mediated by ANG are required [150]. One should also pay attention to the ANG—ribonuclease inhibitor (RNH1) system, which is seen as one of the auxiliary parts of the translation machinery, where RNH1 is regarded as a “sentinel” to protect the RNA from extracellular RNAses [151].

**Figure 1:**
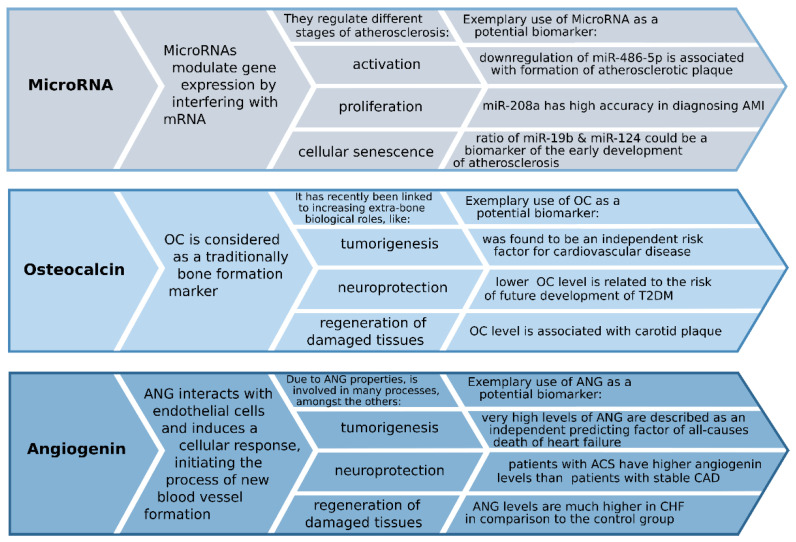
Schematic diagram of potentially new biomarkers mechanism.

In conclusion, ANG is one of the most potent angiogenic factors and appears to play a role both in physiology and pathology, where angiogenesis is involved. A schematic diagram describing the basic information and exemplary use of osteocalcin, MicroRNA, and angiogenin as potentially new biomarkers is presented in Figure 1. Relatively high circulating levels of ANG in healthy individuals indicate an essential physiological role for the molecule. Abnormal ANG levels are commonly seen in cancer, but this molecule has also been linked to other non-malignant diseases, including CVD. Concerning CVD, ANG may have validity in diagnostic and prognostic capacity in ACS, CAD, and CHF. It has been shown as a potential novel marker of CAS [147]. However, the studies of the link between this angiogenic marker and atherosclerosis should be more explored.

A summary of findings about diagnosis and prognosis from the studies mentioned in Section 2.2 concerning potentially new CVD biomarkers can be found in Table 2.

## 3. Limitations

As with the majority of studies, the design of the current study is subject to limitations. A few quoted articles concern animal models, which do not always fully correspond to human physiology. In addition, the homogeneity of the research objects between the articles has not been checked; therefore, some of the mentioned biomarkers can be more or less noticeable for a specific gender or age group. This study reviews the current literature suggesting that these biomarkers can be used in clinical settings; however, some of them, such as miRNAs, require higher resources and long determination times, which can limit their use.

## 4. Conclusions

While all selected biomarkers are promising, further research, both basic and clinical, is needed to understand better their importance in assessing and forming atherosclerotic plaque and its clinical implications after rupture. Finding a suitable biomarker appears tedious and complicated for a number of reasons outlined in this review. However, as molecular research advances, it is essential to understand that we most likely will not find one unique biomarker that could be used for each studied group. Instead, groups of biomarkers related to key pathways identified in atherosclerosis research should be considered. This opens the way for personalized and systems medicine, without which it seems impossible to go any further.

## Figures and Tables

**Table 1:** Summary of findings about diagnosis and prognosis from studies concerning recognized cardiovascular biomarkers.

Study	Biomarker	Findings
Role of C-reactive protein at sites of inflammation and infection [8]	CRP	The existence of two protein conformations—pCRP and mCRP may explain conflicting data on CRP properties.
High sensitivity C-reactive protein (hsCRP) & cardiovascular disease: an indian perspective [12]	CRP	High sensitivity CRP tests help to quantify a low degree of systemic inflammation in the absence of overt systemic inflammatory or immune disorders. HsCRP tests have been standardized on several commercial platforms and can be accurately measured in fresh or frozen plasma.
Markers of inflammation and cardiovascular disease: application to clinical and public health practice: a statement for healthcare professionals from the Centers for Disease Control and Prevention and the American Heart Association [13]	CRP	Considering the hs-CRP results, patients can be assigned to a group of the CVD development risk: low-risk (<1.0 mg/L), intermediate-risk (1.0–3.0 mg/L), and high-risk (>3.0 mg/L).
Gender and C-reactive protein: data from the Multiethnic Study of Atherosclerosis (MESA) cohort [14]	CRP	Women have higher median CRP levels than men; therefore, there is a need for establishing different cutoff values for cardiovascular risk according to gender.
C-reactive protein concentration and risk of coronary heart disease, stroke, and mortality: an individual participant meta-analysis [15]	CRP	CRP has a similar importance as traditional cardiovascular risk factors and other markers of inflammation; CRP level is higher in diabetic patients, alcohol drinkers, and people who are not physically active.
Interleukin-1beta inhibition and the prevention of recurrent cardiovascular events: rationale and design of the Canakinumab Anti-inflammatory Thrombosis Outcomes Study (CANTOS) [16]	CRP	hsCRP has a strong persistence of concentration, even in a population of people with high baseline values.
Role of microcirculatory function and plasma biomarkers in determining the development of cardiovascular adverse events in patients with peripheral arterial disease: A 5-year follow-up [17]	CRP	hs-CRP level is a risk factor for peripheral arterial disease. Its level is related to subclinical carotid atherosclerosis, but such a relationship was not noticed among patients with type 2 diabetes mellitus.
High-sensitivity C-reactive protein as cardiovascular risk marker in patients with diabetes mellitus [19].	CRP	Increasing hs-CRP levels are associated with an increased risk of developing type 2 diabetes mellitus with all levels of metabolic syndrome. hs-CRP levels increase with β cells dysfunction and insulin resistance.
Baseline high-sensitivity C-reactive protein predicts macrovascular and microvascular complications of type 2 diabetes: a population-based study [20].	CRP	Adding hs-CRP to the list of risk factors for vascular events in patients with T2DM improves its predictive value of CHDE and microvascular complications. Implementing hs-CRP is more significant in patients with T2DM.
Relationship of lipids, C-reactive protein and sialic acid in the healthy individuals [21]	CRP	CRP is a predictor of atherosclerosis; there is a statistically significant correlation between CRP, sialic acid, and HDL levels.
Quantification of coronary atherosclerosis and inflammation to predict coronary events and all-cause mortality [22].	CRP	Elevated CAC and hs-CRP levels are indicative of advanced coronary atherosclerosis and systemic inflammation; in their presence, comorbidities appeared to be associated with a higher risk of death.
C-reactive protein modulates risk prediction based on the Framingham Score: implications for future risk assessment: Results from a large cohort study in southern Germany [23].	CRP	CRP and FRS lead to a more accurate prediction of a first coronary event than the results achieved by using those markers separately.
C reactive protein, inflammation and coronary heart disease [27]	CRP	The American Heart Association suggests that when evaluating CVD risk, levels of hs-CRP above 10 mg/L should be discarded as they are probably the reflection of acute inflammation. However, Shrivastava et al. claims that patients with hs-CRP levels above 20 mg/L are, in fact, at the very highest risk.
Comparative analysis of Bayer wide-range C-reactive protein (wr-CRP) and the Dade-Behring high sensitivity C-reactive protein (hs-CRP) in patients with inflammatory bowel disease [28]	CRP	There is a strong correlation between wr-CRP and hs-CRP (for values below 5 mg/L). However, adjustments in the cutoff of the intermediate-risk would be required, because wr-CRP results were substantially lower than those of hs-CRP.
The ability of the wide range CRP assay to classify individuals with low-grade inflammation into cardiovascular risk groups [29]	CRP	Changing the cutoff values between low and moderate risk groups from 1 to 0.9 mg/L helps eliminate differences in group classifications.
High sensitivity C-reactive protein as a cardiovascular risk marker in independent community-living elderly persons [31]	CRP	hs-CRP concentration adds reliable information to the other comparable risk factors among elderly persons.
High-sensitivity C-reactive protein discordance with atherogenic lipid measures and incidence of atherosclerotic cardiovascular disease in primary prevention: the ARIC study [32]	CRP	Patients with higher hs-CRP levels have an independently increased risk of incidental heart failure and all-cause death. hs-CRP levels should be regularly considered together with the measurement of lipids profile in a cardiovascular risk assessment.
High-sensitivity C-reactive protein and hypertension: combined effects on coronary severity and cardiovascular outcomes [33]	CRP	The combination of elevated hs-CRP and hypertension significantly increases the cardiovascular risk in patients with stable, newly diagnosed CAD.
C-reactive protein increase in acute myocardial infarction [34]	CRP	CRP is an indicator of the severity of ST-segment elevation myocardial infarction and can be treated as an indicator of complications during the hospitalization of such patients.
Serum high-sensitivity C-reactive protein levels and the development of atrial fibrillation in a general Japanese population—the Hisayama study [35]	CRP	Serum hs-CRP levels are an independent risk factor for the development of atrial fibrillation in the Japanese population.
Myeloperoxidase (MPO) and high sensitivity C-reactive protein (hsCRP) as inflammatory biomarkers of endothelial and leukocyte activation in overweight hypertensive patients [36]	CRP	hs-CRP is an inflammatory biomarker for overweight, and myeloperoxidase is a biomarker for arterial hypertension.
hsCRP level and the risk of death or recurrent cardiovascular events in patients with myocardial infarction: a healthcare-based study [37]	CRP	hs-CRP was associated with subsequent risk of major adverse cardiovascular events and death.
Multifactorial expression of IL-6 with update on COVID-19 and the therapeutic strategies of its blockade [40]	IL-6	IL-6 levels are associated with dyslipidemia, hypertension, and glucose dysregulation. They are associated with bad outcomes in patients with AMI.
Critical roles of inflammation in atherosclerosis [42]	IL-6	IL-6 is associated with cardiovascular risk in addition to traditional risk factors.There is a correlation between IL-6 levels and endothelial dysfunction and subclinical atherosclerosis; multiple studies indicate that IL-6 signaling plays a role in atherothrombosis.
Inhibition and coronary artery disease in a high-risk population: a prospective community-based clinical study [43]	IL-6	Increased levels of IL-6 are linked to an increased risk of AMI in healthy men. Augmentation in IL-6 and IL-6 receptor levels during the acute phase of AMI is probably related to plaque instability.
Effects of the anti-interleukin-6 receptor antibody, tocilizumab, on serum lipid levels in patients with rheumatoid arthritis [45]	IL-6	Treatments lowering systemic inflammatory markers have a beneficial effect on complications caused by atherosclerosis, but the reduction in inflammatory markers is frequently linked to the mitigation of other risk factors, such as cholesterol levels.
Interleukin-6 receptor pathways in coronary heart disease: a collaborative meta-analysis of 82 studies [46]	IL-6	Asp358Ala polymorphism in the IL-6R gene is a variant that reduces IL-6 signaling, which causes a significant drop in systemic inflammation. Every inherited copy of 358Ala decreases the CAD risk by 3.4% (95% CI, 1.8–5.0).
The interleukin-6 receptor as a target for prevention of coronary heart disease: a mendelian randomisation analysis [47]	IL-6	Asp358Ala polymorphism in the IL-6R gene is a variant that reduces IL-6 signaling, which causes a significant drop in systemic inflammation. Every inherited copy of 358Ala decreases the CAD risk by 5% (95% CI, 3–7).
Exploring potential biomarkers associated with health-related quality of life in patients with coronary artery disease and heart failure [49]	IL-6, CRP	Decreased levels of inflammatory biomarkers, including IL-6 and hs-CRP, were associated with worse health-related quality of life in patients with CAD and heart failure.
Interleukin-6 and the risk of adverse outcomes in patients after an acute coronary syndrome: observations from the SOLID-TIMI 52 trial [50]	IL-6	IL-6 is associated with an increased risk of severe adverse cardiovascular events, including heart failure. IL-6 might be helpful in risk stratification in patients after acute coronary syndrome.
Interleukin-6 as a predictor of the risk of cardiovascular disease: a meta-analysis of prospective epidemiological studies [51]	IL-6	Higher levels of unhealthy people with raised IL-6 were associated with the risk of cardiovascular events over the 7-year average. IL-6 levels were positively associated with hypertension and hypercholesterolaemia but negatively associated with triglyceride levels.
Interleukin-6 gene polymorphism and lipid abnormalities in healthy subjects [52]	IL-6	Circulating IL-6 is associated with higher levels of circulating triglycerides but not total cholesterol levels, which suggests that differential expression of the IL-6 gene may affect the lipid profile.
Antioxidant and inflammatory aspects of lipoprotein-associated phospholipase A₂ [55]	Lp-PLA2	Lp-PLA2 is increased in subjects with the incidence of CVD and in patients with heart failure.
Lipoprotein-associated phospholipase A2 prognostic role in atherosclerotic complications [56]	Lp-PLA2	Lp-PLA2 is one of the most promising atherosclerosis biomarkers that can be useful in assessing cardiovascular risk in asymptomatic patients.
Lipoprotein-associated phospholipase A(2) interacts with phospholipid vesicles via a surface-disposed hydrophobic α-helix [58]	Lp-PLA2	Plasma levels of Lp-PLA2 have been identified as a biomarker of vascular inflammation and atherosclerotic vulnerability, which help predict future cardiovascular events.
Oxidized phospholipids and lipoprotein-associated phospholipase A2 as important determinants of Lp(a) functionality and pathophysiological role [60]	Lp-PLA2	The HDL-Lp-PLA2 level is reduced in patients with combined hyperlipidemia, primary hypertriglyceridemia, pre-diabetes, and metabolic syndrome, while LDL-Lp-PLA2 level is elevated in these patients.
Association between high-sensitivity C-reactive protein, lipoprotein-associated phospholipase A2 and carotid atherosclerosis: A cross-sectional study [61]	Lp-PLA2	There are inconsistencies regarding whether this lipase can be used to predict atherosclerosis. As a result of this, it may be helpful to use a combination of hs-CRP and Lp-PLA2 to predict the risk of CVD, including CAD and stroke.
Lipoprotein-associated phospholipase A2 and risk of coronary disease, stroke, and mortality: collaborative analysis of 32 prospective studies [62]	Lp-PLA2	Activity and mass of Lp-PLA2 show a continuous relationship with the risk of coronary heart disease, similar in magnitude to non-HDL cholesterol or systolic blood pressure in the studied population. However, researchers noted that circulating Lp-PLA2 in the blood may be an imperfect indicator of its importance in atherosclerotic plaque.

AMI: acute myocardial infarct; CAC: coronary artery calcification; CAD: coronary artery disease; CHDE: coronary heart disease events; CVD: cardiovascular diseases; HDL: high density lipoprotein; T2DM: type 2 diabetes mellitus

**Table 2:** Summary of findings about diagnosis and prognosis from studies concerning potentially new CVD biomarkers.

Study	Biomarker	Findings
Impact of miRNA in atherosclerosis [68]; Analysis coupled with text mining identify novel biomarker candidates for recurrent cardiovascular events [69]	miR-21	miR-21 expression was increased in peripheral blood mononuclear cells (PBMCs) in patients with severe vascular disease and AMI in the medical history.
Regulation of microRNAs in coronary atherosclerotic plaque [72]	miR-486-5p	Formation of atherosclerotic plaque is associated with miR-486-5p downregulation.
Circulating miR-146a may be a potential biomarker of coronary heart disease in patients with subclinical hypothyroidism [73]	miR-146a	miR-146a is a possible predictor of atherosclerosis and its severity in patients with hypothyroidism and raised TSH level.
MiR-664a-3p expression in patients with obstructive sleep apnea: A potential marker of atherosclerosis [75]	miR-664a-3p	In patients with increased maximum carotid intima-media thickness and obstructive sleep disorder, levels of miR-664a-3p are raised.
Circulating microRNAs identify patients at increased risk of in-stent restenosis after peripheral angioplasty with stent implantation [76]	miR-195	miR-195 level decreases in patients with increased chance of possible adverse ischemic events after stenting.
Expression profiles of six atherosclerosis-associated microRNAs that cluster in patients with hyperhomocysteinemia: a clinical study [78]	miR-217	In patients with hyperhomocysteinemia and atherosclerosis, upregulation of miR-217 is much higher.
miR-145 and miR-143 regulate smooth muscle cell fate and plasticity [80].Plasma miR-126 and miR-143 as potential novel biomarkers for cerebral atherosclerosis [81]	miR-126, miR-143	Downregulation of miR-126 and miR-143 may be a valuable predictor of cerebral atherosclerosis in patients.
Circulating microRNAs as biomarkers of disease and typification of the atherothrombotic status in antiphospholipid syndrome [83]	miR-19b, miR-124	Ratio of miR-19b and miR-124 could be a biomarker of the early development of atherosclerosis.
A common variant in pre-miR-146 is associated with coronary artery disease risk and its mature miRNA expression [85]	miR-146a, miR-146b	In patients with CAD increased expression of miR-146a or miR-146b correlates with an increased risk of atherosclerosis.
Micro RNA expression profile of human advanced coronary atherosclerotic plaques [86]	miR-21, miR-221, miR-222	miR-21 could be a potential biomarker for distinguishing symptomatic plaque burden.miR-221 and miR-222 are anti-atherosclerotic miRNAs which can be used as diagnostic and prognostic biomarkers in atherosclerosis and inflammatory diseases.
Significant association between functional microRNA polymorphisms and coronary heart disease susceptibility: a comprehensive meta-analysis involving 16,484 subjects [87]	microRNA-146a rs2910164, microRNA-196a2 rs11614913, microRNA-499rs3746444	CVD risk is associated with microRNA-146a rs2910164 polymorphism, microRNA-196a2 rs11614913 and microRNA-499rs3746444 exhibit similar associations.
MicroRNA-133a and myocardial infarction [89]	miR-1, miR-133a, miR-208a/b, miR-499	miR-1 miR-133a, miR-208a/b and miR-499 are upregulated and increase in circulation shortly after AMI.
Circulating microRNA: a novel potential biomarker for early diagnosis of acute myocardial infarction in humans [90]	miR-208a	miR-208a has high accuracy in diagnosing AMI—it is increased in one hour after occlusion in 90% of AMI patients, and in 100% of AMI patients within four hours.
Plasma miR-1, miR-208, miR-499 as potential predictive biomarkers for acute myocardial infarction: An independent study of Han population [91]	miR-499	miR-499 have higher predictive value and reliability than biomarkers such as TnT and CKMB.
Analysis of plasma miR-208a and miR-370 expression levels for early diagnosis of coronary artery disease [92]	miR-208a, miR-370	Combination of miR-208a and miR-370 are promising diagnostic biomarkers for CAD diagnosis.
Overview of 8 circulating MicroRNAs and their functions as major biomarkers for cardiovascular diseases [93]	miR1, miR-21, miR-126, miR-133, miR-145, miR-208, miR-223, miR-499	miR1, miR-21, miR-126, miR-133, miR-145, miR-208, miR-223, and miR-499 have been recognized as CAD biomarkers with significant clinical value.
Aspirin treatment hampers the use of plasma microRNA-126 as a biomarker for the progression of vascular disease [94]	miR-126	miR-126 levels were decreased in patients with AMI after the onset of symptoms; aspirin reduces circulating miR-126 levels.
Plasma levels of microRNA-145 are associated with severity of coronary artery disease [95]	miR-145	In CAD patients, miR-145 levels were decreased; it is associated with the severity of CAD.
Diagnostic and prognostic value of circulating microRNAs in patients with acute chest pain [96]	miR-208b	miR-208 levels are increased in CAD and are associated with mortality of CADs. miR-208b could be one of the mortality-predicting biomarkers for AMI after adjustment for age and sex.
miRNA-197 and miRNA-223 predict cardiovascular death in a cohort of patients with symptomatic coronary artery disease. [97]	miR-223	miR-223 is higher in CAD patients and associated with increased mortality and an increased risk for future AMI.
Association of circulating osteocalcin with cardiovascular disease and intermediate cardiovascular phenotypes: systematic review and meta-analysis [112]	OC	OC is associated with atherosclerotic changes—it may be a specific arteriosclerosis marker in the future. It was found to be an independent risk factor for cardiovascular disease.
Low serum osteocalcin concentration is associated with incident type 2 diabetes mellitus in Japanese women. [121]	OC	Risk of the future development of T2DM was found to be related to lower circulating OC level in Japanese postmenopausal women.
Serum osteocalcin level and its association with carotid atherosclerosis in patients with type 2 diabetes [123].Relationship between serum osteocalcin levels and carotid intima-media thickness in Chinese postmenopausal women. [124]	OC	In T2DM patients with carotid atherosclerosis in T2DM [123] and Chinese postmenopausal woman [124], low levels of OC have been found.
Serum levels of osteocalcin in relation to glucose metabolism and carotid atherosclerosis in Chinese middle-aged and elderly male adults: the Shanghai Changfeng Study [126]	OC	OC level is associated with carotid plaque even after adjusting for CVD risk factors in euglycemic patients.
Osteocalcin and abdominal aortic calcification in hemodialysis patients: an observational cross-sectional study [129]	OC	OC proved to be a risk factor for vascular calcification, but further research is required to determine if ucOC is directly related to vascular calcification.
Association between the circulating total osteocalcin level and the development of cardiovascular disease in middle-aged men: a mean 8.7-year longitudinal follow-up study [134]	OC	Serum total OC level was not associated with the development of CVD after adjusting for other CVD risk factors.
Ethnic differences in osteocalcin γ-carboxylation, plasma phylloquinone (vitamin K1) and apolipoprotein E genotype [135]	OC	There may be differences in circulating OC levels between the various ethnic groups.
Circadian rhythm of osteocalcin in the maxillomandibular complex [136],	OC	Time of blood sampling may affect the variability of the OC results, because of its circadian rhythm.
The role of vitamin K in humans: implication in aging and age-associated diseases [137]	OC	Vitamin K and vitamin D influence should be reconsidered before OC becomes a recognized cardiovascular biomarker.
Plasma angiogenin levels in acute coronary syndromes: implications for prognosis [144]	ANG	Patients with ACS have higher angiogenin levels than patients with stable CAD and a healthy control group.
Prognostic significance of angiogenic growth factor serum levels in patients with acute coronary syndromes [147]	ANG	Patients with CAD have significantly higher ANG concentration than the control group; ANG is increased in patients with higher Gensini scores.
Matrix metalloproteinases: a review of their structure and role in acute coronary syndrome [148]	ANG	ANG levels above 426 ng/mL have been described as an independent predicting factor of all-cause death of heart failure with preserved ejection fraction.
Elevated angiogenin levels in chronic heart failure [149]	ANG	ANG levels were much higher in CHF than compared to the control group; elevated ANG levels were predictive of adverse events such as death.
